# Sense of smell in chronic rhinosinusitis: A multicentric study on 811 patients

**DOI:** 10.3389/falgy.2023.1083964

**Published:** 2023-04-21

**Authors:** Alberto Macchi, Alessia Giorli, Elena Cantone, Giorgia Carlotta Pipolo, Flavio Arnone, Umberto Barbone, Giacomo Bertazzoni, Chiara Bianchini, Andrea Ciofalo, Federica Cipolla, Alessio De Massimi, Carla De Vita, Cristina Di Lieto, Angelo Ghidini, Marco Govoni, Giulia Gramellini, Alessandro Maselli Del Giudice, Giancarlo Ottaviano, Veronica Seccia, Federico Sireci, Giacomo Sollini, Claudia Staffieri, Stefania Gallo, Enrico Heffler, Ignazio La Mantia, Eugenio De Corso, Frank Rikki Canevari, Nicola Lombardo, Luca Malvezzi, Gabriele Orietti, Ernesto Pasquini, Livio Presutti, Giulia Monti

**Affiliations:** ^1^ ENT Clinic Asst Sette Laghi – University of Insubria, Varese, Italy; ^2^ ENT Unit, University of Siena, Siena, Italy; ^3^ Department of Neuroscience, Reproductive and Odontostomatologic Sciences, Unit of Ear, Nose and Throat, Federico II University, Naples, Italy; ^4^ Otorhinolaryngology Unit ASST Santi Paolo e Carlo Hospital. Department of Health Sciences, University of Milan, Milan, Italy; ^5^ ENT Department, Ospedale Provinciale Bolzano, Bolzano, Italy; ^6^ Ent Clinic, Asst Cremona, Cremona, Italy; ^7^ ENT Department, Azienda Ospedaliera Universitaria S.Anna, Ferrara, Italy; ^8^ Organ of Sense Department, Sapienza University of Rome, Roma, Italy; ^9^ ENT Clinic, University of Catania, Catania, Italy; ^10^ SS ORL San Vito al Tagliamento - Azienda Sanitaria Friuli Occidentale, S. Vito al Tagliamento, Italy; ^11^ Otolaryngology and Audiology Unit, IRCCS Azienda Ospedaliero Universitaria Policlinico di Sant-Orsola, Bologna, Italy; ^12^ ENT Department, Arcispedale Santa Maria Nuova, Reggio Emilia, Italy; ^13^ ENT Department, University of Parma, Parma, Italy; ^14^ UOC Otorinolaringoiatria, ASST Grande Ospedale Metropolitano Niguarda, Milano, Italy; ^15^ ENT Unit Bari Adria Trani, Bari, Italy; ^16^ ENT Clinic, Azienda Università Padova, Padova, Italy; ^17^ ENT Clinic, University of Pisa, Pisa, Italy; ^18^ Otorhinolaryngology Section, Department of Biomedicine, Neuroscience and Advanced Diagnostics (BIND), University of Palermo, Palermo, Italy; ^19^ ENT Unit Metropolitan Area, AUSL Bologna, Bologna, Italy; ^20^ ENT Clinic Treviso, Treviso, Italy; ^21^ IRCCS Humanitas Research Hospital, Humanitas University Rozzano, Milano, Italy; ^22^ Humanitas University, Pieve Emanuele (MI), Milano, Italy; ^23^ Istituto di ORL, Fondazione Policlinico Universitario A. Gemelli IRCCS, Roma, Italy; ^24^ Unit of Otorhinolaryngology - IRCCS Ospedale Policlinico San Martino, Genova, Italy; ^25^ UOC Azienda Ospedaliero Universitaria Mater Domini, Catanzaro, Italy; ^26^ ENT Clinic, University of Insubria, Varese, Italy

**Keywords:** CRSwNP, smell, type 2, QoL (quality of life), olfactory dysfunction

## Abstract

**Introduction:**

The impairment of the sense of smell is often related to chronic rhinosinusitis (CRS) with or without nasal polyps (CRSwNP, CRSsNP). CRSwNP is a frequent condition that drastically worsens the quality of life of those affected; it has a higher prevalence than CRSsNP. CRSwNP patients experience severe loss of smell with earlier presentation and are more likely to experience recurrence of their symptoms, often requiring revision surgery.

**Methods:**

The present study performed a multicentric data collection, enrolling 811 patients with CRS divided according to the inflammatory endotype (Type 2 and non-Type 2). All patients were referred for nasal endoscopy for the assessment of nasal polyposis using nasal polyp score (NPS); Sniffin’ Sticks olfactory test were performed to measure olfactory function, and SNOT-22 (22-item sinonasal outcome test) questionnaire was used to assess patients’ quality of life; allergic status was evaluated with skin prick test and nasal cytology completed the evaluation when available.

**Results:**

Data showed that Type 2 inflammation is more common than non-type 2 (656 patients versus 155) and patients suffer from worse quality of life and nasal polyp score. Moreover, 86.1% of patients with Type 2 CRSwNP were affected by a dysfunction of the sense of smell while it involved a lesser percentage of non-Type 2 patients. Indeed, these data give us new information about type-2 inflammation patients’ characteristics.

**Discussion:**

The present study confirms that olfactory function weights on patients’ QoL and it represents an important therapeutic goal that can also improve patients’ compliance when achieved. In a future – and present – perspective of rhinological precision medicine, an impairment of the sense of smell could help the clinician to characterize patients better and to choose the best treatment available.

## Introduction

Chronic rhinosinusitis (CRS) in adults is defined as a chronic inflammatory disease involving nasal mucosa and paranasal sinuses associated with long-term symptoms such as nasal blockage, obstruction, congestion, or nasal discharge and facial pain/pressure and reduction or loss of smell, lasting 12 weeks or longer (1). The prevalence of CRS occurs in >10% of the adult population in Europe and the United States; in particular, the one with nasal polyps (CRSwNP) estimates for 5% of the population, and it is associated with significant morbidity and reduced health-quality of life (QoL) (2–4). Nowadays, CRS is clinically differentiated into two phenotypes: CRS without nasal polyps (CRSsNP) and CRSwNP with a different predominance of symptoms and inflammation. Inflammatory pathways actually determine the CRS endotype to be divided into type 2 and non-type 2. In the literature, most patients with CRSwNP show evidence of type 2 airway inflammation rather than patients afflicted by CRSsNP. Indeed, type 2 endotype in CRSwNP and non-type 2 CRSsNP are two mainstays of a wide scenery. Type 2 inflammation presents the involvement of both innate and adaptive immune systems. In particular, it presents high levels of type 2 innate lymphoid cells (ILC2s) and Th2 helper cells, and it is mediated by IL-4, IL-5, and IL-13 cytokines. It is characterized by tissue eosinophilia and high IgE levels. Non-type 2 CRS, instead, is related to Th1/Th17-mediated immune responses and it is characterized by cytokines such as IL-17A, IL-8, interferon-gamma (IFN-*γ*), and, in particular, by neutrophilic inflammation (5). Since type 2 inflammation is involved in the pathogenesis of other comorbidities such as asthma, it determines clinical major disease severity and greater morbidity than non-type 2 inflammation (6–8). Typically patients with type 2 inflammation require a higher number of surgeries and need numerous medical treatments (3). Characteristic symptoms of both CRS are similar but with different prevalences in each one. The impairment of the sense of smell is one of the main complaints reported by patients with CRSwNP and it may be considered one of the first signs of disease recurrence (9, 10). It consists of one of the most bothersome symptoms and its importance is proved by its insertion as one of the four symptoms to clinically diagnose CRS in the American and European rhinosinusitis guidelines (1, 11). Olfactory dysfunction affects almost 67%–78% of CRS patients, and nowadays it is a subject of extreme interest among researchers (12). Despite its significance being well-defined, the mechanism of smell impairment in CRS is still quite unclear, even if considered an inflammatory cause. Generally, volatile odorants reach the olfactory epithelium at the level of the cribriform plate, the upper part of the nasal septum, and the middle/upper turbinate, and then dissolve into the mucus layer to activate olfactory receptors. In CRS patients, this mechanism is subverted, and few hypotheses try to explain the possible mechanism. According to the sensorineural hypothesis, the chronic inflammation of the neuroepithelium and the edema on the olfactory mucosa contribute to decreasing the transmission of synaptic olfactory impulses. However, the conductive hypothesis states that the change in the airflow due to the presence of nasal polyps or edematous mucosal tissue could be responsible for the olfactory impairment (13). Indeed, smell loss is also feasible in patients without obstruction or altered airflow and, on the other side, in many patients, the removal of nasal polyps does not improve the olfactory function (14). The common basis of these theories is that the inflammatory process of CRS plays a key role in the patient's olfactory dysfunction.

Olfactory impairment has been described as a major symptom of CRSwNP affecting 83%–91% of patients and it seems to be more severe and frequent, and with earlier expression in patients with eosinophilic infiltration, usually linked to type 2 inflammation compared to CRSsNP patients (12, 15, 16). The loss of smell is worse in the earlier stage of CRS, particularly due to eosinophilic infiltration in blood and nasal mucosa. As proof of that, the Charcot–Leyden crystal (CLC) gene expression, a marker of eosinophilic infiltration, is significantly correlated with the olfactory threshold. It appears that the severity of smell loss could have a role as a surrogate marker of inflammation in all the nasal mucosa. Furthermore, in patients affected by CRS and smell dysfunction, many histologic changes are reported including goblet cell hyperplasia, squamous metaplasia, infiltration of inflammatory cells, and olfactory epithelial layer loss. Peripheral inflammation harms the mucus layer of the respiratory and olfactory epithelium. Generally, the mucus is produced by the respiratory mucosa and Bowman's gland. The inflammation may lead to hypersecretion and altered concentration of potassium and sodium, modifying the olfactory mucus and may interfere with olfactory receptor activation (17). These peripheric changes that occur in the nasal mucosa could have consequences even on the central system. It is proven that in patients with CRS, a reduction in the olfactory bulb volume could occur (18). The reduction of the bulb could happen because it directly receives axons from the olfactory epithelium. In CRS patients, it receives a decreased input from the peripheric inflamed area, causing its dimension reduction. The inflammatory state in the nasal cavity is probably sufficient to produce a gradual and cumulative effect in central areas, which may contribute to smell loss in CRS patients. Despite that, it remains unknown which specific processes are responsible for these changes in the central nervous system. Therefore, olfaction is taking space as a marker of the degree of inflammation in the CRS panorama, and as a possible marker to differentiate CRS phenotypes and endotypes. Nevertheless, little is still known about the various changes and aspects it could display in patients affected by type 2 and non-type 2 rhinosinusitis. Given the importance of the role of olfaction in the CRS panorama and its relationship with inflammation, in this paper, we aim to evaluate olfactory dysfunction with particular regard to its clinical features in patients affected by type 2 and non-type 2 chronic rhinosinusitis.

## Materials and methods

The current study is a multicenter retrospective real-life observational study focused on an educational program about smell disorders, especially the ones in type 2 inflammation. The data came from 25 Italian ENT departments whose investigators were trained and instructed to perform all the analyses included in the study. The program is authorized by the Italian Agency of the Health Ministry (AGENAS) under the program of Continuous Education in Medicine (ECM).

All the participants were patients who accessed a referred ENT clinic for nasal complaints in 2019. The inclusion criteria were the presence of rhinosinusitis that was diagnosed based on the validated criteria defined by The European Position Paper on Rhinosinusitis and Nasal Polyps (EPOS 2020) (1). The exclusion criteria were cases of common cold, allergic rhinitis, neoplastic pathology, and lack of informed consensus.

All patients provided written informed consent and underwent different diagnostic procedures performed to evaluate the function of smell and nasal inflammation status. The ENT specialist of each ENT clinic referred the patient’s to accurate medical history, nasal endoscopy to collect Nasal Polyp Score (NPS), 22-item Sinonasal Outcome Test questionnaire (SNOT-22), Sniffin’ Sticks Identification Smell Test, the Skin Prick Test, and nasal cytology where available. Type 2 inflammation was determined with laboratory tests for circulating biomarkers: immunoglobulin (IgE) and eosinophils (EOS). We referred to EPOS2020 for type 2 inflammation cut-offs: patients were included in the type 2 group when showing peripheral blood eosinophilic count ≥250 cells/mm^3^ or total IgE ≥100 kU/l (1). Clinical medical history was considered, taking into account the number of CRS exacerbations. Nasal polyp status was assessed by bilateral nasal endoscopy with a 2.7 mm 30° rigid endoscope (Karl Storz, Tuttlingen, Germany). Polyp size was evaluated endoscopically using NPS. The scale ranges from 0 (no polyp) to 4 (large polyps) for each nostril and the total score ranges from 0 to 8; higher scores indicate worse condition (4). To determine the quality of life, all the patients were submitted to SNOT-22. The SNOT-22 is a disease-specific questionnaire, widely used as a patient-reported outcome that evaluates the impact of CRS on a patient's quality of life with a recall period of 2 weeks. The questionnaire is composed of 22 CRS-related items each one scored from 0 to 5, for a total score range of 0–110 with higher scores representing worse conditions (19, 20). As regards allergy, a skin prick test was performed to establish the patient's allergic status. It consisted of pricking the skin on the volar surface of the forearm, with a lancet through a drop of allergen extract following the international guidelines of the prick test (21). Finally, the sense of smell was assessed using Sniffin’ Sticks—16 items identification test (SS-I) (Burghart instruments, Wedel, Germany). The identification test is part of the Sniffin’ Sticks Smell Test, a standardized test for the assessment of smell dysfunction. It is based on 16 common odors, each one presented in a forced multiple choice from a list of four items (three distractors and one target) (22). A normal smell function is considered when the patient scores ≥12 correct answers out of 16. Patients with scores from 9 to 11 are considered hyposmic and patients with scores ≤8 are defined as anosmic. Finally, nasal cytology was performed in ENT centers where it was already available before the present study was started. Cytological sampling consists of the collection of superficial cells from the nasal mucosa of the middle portion of the inferior turbinate, performed either with the aid of a sterile swab or by use of a small curette (scraping) in disposable plastic material (Nasal scraping®—EP Medica, Fusignano, Italy). The sampling is stained with May–Grünwald–Giemsa (MGG) and read at optical microscopy with a 1000× objective with immersion oil. Fifty fields of each specimen were read for each sample and the count of each cell type (ciliated cells, goblet cells, eosinophils, mast cells, neutrophils) was performed using a semiquantitative grading, as suggested by recent evidence (23). The data were analyzed in each center and then collected as a cumulative work. Statistical analysis was performed using descriptive methods.

## Results

Twenty-five centers participated in the educational program. The number of patients enrolled varied from center to center, and this was due to pandemic restrictions for COVID. Only four centers did not send the data in time. The number of patients enrolled was different for each center and the total number of patients who were enrolled in this study is 811.

This is a nonrandomized observational study based on 811 patients affected by CRSwNP. Patients were divided into two groups considering those affected by type 2 CRSwNP and non-type 2 CRSwNP.

From the overall number of patients, patients in the type 2 group were 656 (80.9%) while patients in the non-type 2 group were 155 (19.1%).

Considering the different numbers of patients in the two groups, we compared the demographics (also summarized in Table 1). The mean age of patients in both groups was 52.3 years old, with no statistically significant difference between groups (*p* = 1).

**Table 1:** A brief description of the population included in the study, divided in the two groups (type 2/non-type 2).

	All patients (*n* = 811)	Type 2 (*n* = 656, 80.9%)	Non-type 2 (*n* = 155, 19.1%)	*p*-value
Gender	* *	* *	* *	*0.9194*
Male (%)	482 (59.4)	385 (58.7)	97 (61.6)	* *
Female (%)	329 (40.6)	271 (41.3)	58 (38.4)	* *
Age	52.3	52.3	52.3	*1*
Allergic comorbidities (%)	445 (54.9)	398 (60.7)	37 (23.9)	*<0.0001*
Peripheral eosinophils (mean value, cells/mm^3^)	724.6	761.4	184.1	*<0.0001*
SNOT-22 (mean value)	45.4	47.5	36.3	*<0.0001*
NPS (mean value)	4.3	4.6	3.0	*<0.0001*
NPS male	4.5	4.6	3.1	* *
NPS female	4.0	4.6	2.9	* *
Previous surgery (%)	136 (16.8)	133 (20.3)	3 (1.9)	*<0.0001*

NPS, nasal polyp score; SNOT-22, 22-item Sinonasal Outcome Test questionnaire.

*p*-value from statical analysis: we considered a significant difference for *p* values <0.05.

Considering all patients, 466 (59.4%) were males and 345 (40.6%) were females. In the type 2 group, 385 (58.7%) were males and 271 (41.3%) were females, while in the non-type 2 group, there were 97 (61.6%) males and 58 (38.4%) females. There is no statistically significant difference between the two groups (*p* = 0.9194).

*Allergic status.* The allergic comorbidities were present in 445 out of 811 (54.9%) patients. In type 2 groups, 398 (60.7%) were allergic, and in the non-type 2 group, only 37 (23.9%) were allergic.

*Endoscopic evaluation.* Regarding the nasal polyp score, our results show a cumulative NPS mean value of 4.3, with 4.5 as the NPS mean value in males and 4.0 in females. In type 2 patients, the mean value of NPS was 4.6, while in non-type 2 patients, it was 3.0. Considering patients’ sex, male patients showed a mean NPS of 4.6 in the type 2 group, and 3.1 in the non-type 2 group. Females with type-2 had a mean NPS of 4.6, and in those with non-type 2, it was 2.9.

*Quality of life.* The 22-item Sinonasal Outcome Test showed an overall mean value of 45.4, but different results were observed between the type 2 (SNOT-22 mean value: 47.5) and the non-type 2 groups (mean value: 36.3).

*Smell identification test.* Performing the Sniffin’ Sticks Identification Test, we considered a score greater or equal to 12 out of 16 as a normal smell function. Patients with scores ranging from 8 to 11 were considered hyposmic and score less than 8 out of 16 were classified as anosmic.

Our data show an overall number of 675 (83.2%) patients with impaired olfactory function, while 136 (16.8%) had a normal sense of smell.

In the type 2 group, patients showed smell dysfunction in 565 cases (86.1%) and 91 patients showed normal results (13.9%). In the non-type 2 group, 110 patients (71.0%) had smell impairment while 45 (29.0%) had no olfactory dysfunction. Different results divided according to patients’ sex are shown in Table 2.

**Table 2:** Data from the Sniffin’ Sticks Identification Test divided in the two groups and showing differences between different percentage of smell impairment between males and females.

Smell identification test	Type 2 group (*n* = 656)	Non-type 2 group (*n* = 155)	*p*-value
Mean value (number of correct answers out of 16)	6.4	8.5	* *
Smell impairment, no. of patients (%)	565 (86.1)	110 (71.0)	*0.0042*
Male	327 (84.9)	72 (74.2)	* *
Females	238 (87.8)	38 (65.5)	* *
Normal values, no. of patients (%)	91 (13.9)	45 (29.0)	*0.0042*
Male	58 (15.1)	25 (25.8)	*** ***
Females	33 (12.2)	20 (34.5)	*** ***

*p*-value from statical analysis: we considered a significant difference for *p* values <0.05.

**Figure 1:**
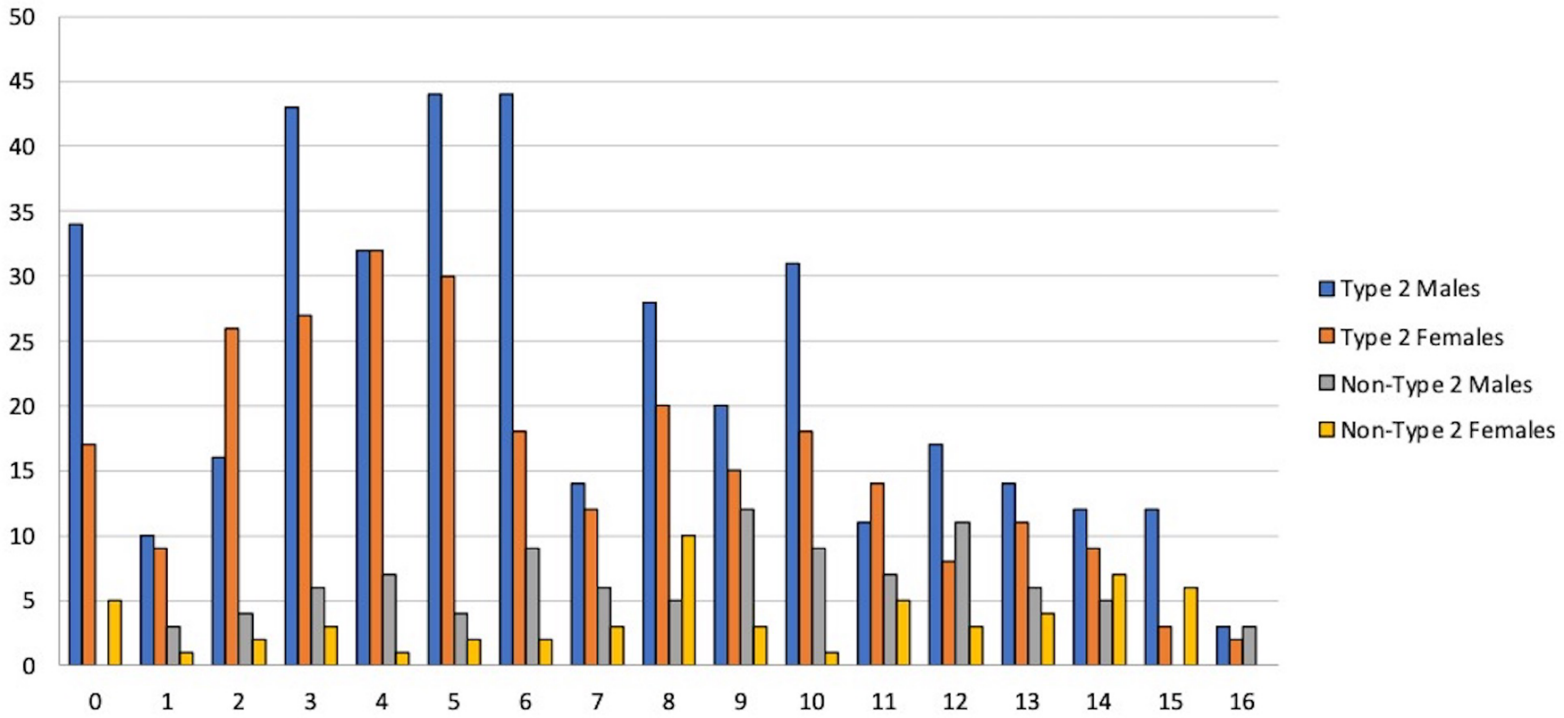
Sniffin’ Sticks Identification Test: distribution of total scores in male and females in both type 2 and non-type 2 groups. Patients were enrolled in 21 different Hospital Centers in Italy: 109 in Varese, 101 patients in Bologna (AUSL), 73 in Genova, 67 in Milano (Ospedale San Paolo), 62 in Rome (Policlinico Gemelli), 51 in San Vito al Tagliamento, 49 in Catania, 43 in Napoli (University Federico II), 39 in Pisa, 30 in Milano (Humanitas Hospital), 25 in Padova, 21 in Catanzaro, 20 in Bologna (Policlinico Sant’Orsola), 20 in Cremona, 20 in Palermo, 20 in Pavia, 16 in Milano (Ospedale Niguarda), 14 in Parma, 13 in Bolzano, 10 in Barletta, and 8 in Treviso.

Mean value of the smell identification test was 6.4 out of 16 in the type 2 group and 8.5 out of 16 in the non-type 2 group. The distribution of scores of the identification test divided into males and females is shown in Figure 1.

*Nasal cytology*. Nasal scraping was performed only in 453 patients in the type 2 group, corresponding to 55.86% of the overall number of patients enrolled and to 69.05% of patients in the type 2 group. This examination was considered not mandatory in this study, but it can be useful to understand how the nasal cellularity of patients may characterize at least one type of CRSwNP and the severity of the pathology itself. It was performed only in those centers that already performed nasal cytology in their clinical practice before the present study started.

We show the data obtained in the rhinocytograms in Table 3.

**Table 3:** Nasal cytology results.

Nasal cells	Patients (*n* = 453)	Value	%
Eosinophils	398	++++: 295 +++: 55 ++: 40 +: 18	74.0% 13.5% 9.0% 3.5%
Mast cells	54	++: 45 +: 9	
Neutrophils	11	++: 11	
No inflammatory cells	55		
HSS	258	64.8%	
Biofilm	98	24.6%	

HSS, hypercromatic sovranuclear stria; HPFs, high power fields

++++: >20 cells/HPF; +++: 16–20 cells/HPF; ++: 6–15 cells/HPF; +: 1–5 cells/HPF, considering a mean of cells per 50 HPF. The absence of HSS is a sign of loss of functional integrity of ciliated cells.

## Discussion

Our groups show a considerably different number of patients: a higher number of patients were enrolled in the type 2 group. This is reasonable considering that type-2 CRSwNP has a higher prevalence than non-type 2 forms, and our study was performed with nonrandomized data collection and patients were enrolled into case series in different centers (24, 25). Demographically, the two groups show a similar distribution between sexes and a mean age without significant differences. Allergic conditions and peripheral eosinophilia show a significant difference between the two groups, but these data are consistent with the definition of type 2 and non-type 2 inflammation statuses.

Nowadays, olfactory perception is considered one of the most important goals in the treatment of rhinosinusitis as it correlates with the response to medical and surgical treatment of the patient, especially. It also has a great impact on the patient's QoL. Especially in severe patients, smell disorders have become an important clinical feature to be assessed to choose the best treatment modality for each patient. Smell impairment is indeed one of the criteria needed to determine the need for biological therapy in CRSwNP, according to current guidelines (1, 26).

Type 2 inflammation is an inflammatory pathway involving both innate and adaptive immune systems (27). ILC2s and a subpopulation of CD4+ T cells known as Th2 cells can secrete IL-4, IL-5, and IL-13 and stimulate type 2 immunity with high IgE antibodies and eosinophilia. Eosinophils, mast cells, basophils, Th2 cells, ILC2s, and IgE-producing B cells are mediated by the type 2 immune responses. IL-33 and IL-25 regulate maturation of CD4+ T cells into Th2 cells and overproduction of type 2 cytokines (IL-4, IL-5, and IL-13).

Type 2 cytokines drive a cascade of downstream events, including the activation of airway epithelial cells, mast cells, eosinophils, and basophils. Relevant remodeling changes are in smooth muscle cells, mucus cell changes, and vascular remodeling, which predispose the airway mucosa to an exaggerated response to allergens, and environmental stimuli such as viruses, cigarette smoke, and other air pollutants (5, 28).

Eosinophils are one of the most relevant inflammatory cells involved in sustaining the disease. Nasal mucosa accumulation of activated eosinophils is a hallmark of this condition. Furthermore, the percentage of circulatory eosinophils and the prevalence of asthma complications are reported to be significantly higher in patients in the type 2 group than in those in the non-type 2 group.

The inflammatory status seems to have a role in smell impairment as well as the obstructive condition determined by the presence of nasal polyps, for example, altering mucus composition that can make odorants’ conduction more difficult (29).

In patients with CRSwNP, the principal mechanism of the impairment of olfactory function is the obstructive condition caused by the presence of polyps with alteration of nasal airflow and less access of odorants to the olfactory epithelium. This consideration could explain why patients with CRSwNP have worse olfactory ability than those with CRSsNP. Moreover, other evidence may indicate the role of chronic inflammation on olfactory impairment. Inflammatory mediators may disrupt the olfactory neuroepithelium with changes in the transduction of stimuli (30). However, a clear correlation between endotypes and olfactory loss is still to be established or clarified.

As a primary objective, our study evaluated the different prevalence of smell disorders between type-2 and non-type 2 patients. The use of Sniffin’ Sticks is well standardized in patients with olfactory dysfunction, and with the identification test, we evaluated both groups and found a different rate of smell disorders. Both types of CRSwNP show a certain degree of olfactory defect in the identification of the correct answer, but type-2 CRSwNP showed a lower percentage of correct answers, which represents a more compromised olfactory capability. This difference resulted to be significant.

As for the nasal polyp score, it is an important study in patients with type 2 inflammation than in the other group. This is probably correlated with the presence of inflammatory elements at the blood and mucosal levels, and therefore with a greater inflammatory substrate or a higher degree of severity.

A higher NPS may also mean a diminished nasal airflow patency that could contribute to a reduced olfactory capability, but the obstructive mechanism does not seem to fully explain the impairment of smell in CRS (17).

Second, we evaluated the subjective quality of life in the two groups using the 22-item Sinonasal Outcome Test. Our results showed a significantly more compromised QoL in patients in the type 2 group compared to those in the non-type 2 group. It may be interesting to further study the impact of smell disorders in this different impact of CRSwNP in patients’ daily life.

The role of nasal cytology in the definition of type-2 inflammation is still underused and nonstandardized (31). However, our clinical practice usually provides a cytological evaluation in most of the centers involved in this study: nasal cytology allows the definition of predominant cells of the nasal inflammatory infiltrate with possible diagnostic and prognostic implications (32). The samples were read by a semiquantitative reading (33). The most relevant cell type in our findings was the eosinophil as a characteristic of type 2 patients. The mast cells are a sign of local inflammation; some patients have mast cells alone, and 10% of the cases had mast cells with eosinophils. The presence of neutrophils is minimal. The presence of the hyperchromatic supranuclear stria (HSS) is only in 35.2% of the patients who underwent nasal cytological examinations: this is an expected result considering that HSS is usually a marker of loss of functional integrity of the ciliated cell (34). We have found the presence of biofilms in 24.6% of the patients, which is often found in chronic rhinosinusitis.

The study conducted has allowed us to have a detailed background of type 2 and non-type 2 patients, especially focusing on the different impairments of smell. The recent introduction of biological therapies in the treatment protocols of CRSwNP has opened a new perspective on the role of olfaction, both as a diagnostic tool and therapeutic endpoint. The role of endotype may be fundamental to defining the level of impairment of olfactory function. The definition of different expected levels of olfactory impairment in different endotypes of CRS may help the clinician to better understand the characteristics of these patients, leading to more precise diagnosis and treatment.

## Conclusions

This educational program demonstrates how important a correct analysis of patients with CRS and how type 2 inflammation can change clinical presentation.

A complete rhinological evaluation nowadays cannot rule out the assessment of the endotype and the identification of the inflammation in each patient help us to understand the specific need for each one. Olfactory function weights on patients’ QoL, and it represents an important therapeutic goal that can also improve patients’ compliance when achieved.

The assessment of the sense of smell in patients with CRSwNP is important and its impairment is likely to have a relationship with the endotype of patients.

In a future—and present—prospective of a rhinological precision medicine, a better characterization of smell disorders may help the clinician to characterize patients and choose the best treatment available.

## Data Availability

The datasets presented in this study can be found in online repositories. The names of the repository/repositories and accession number(s) can be found in the article/Supplementary Material.
